# Novel orthotopic patient-derived xenograft model using human pancreatic cancer tissue fragments to recapitulate distant metastasis and cancer-related hypercoagulability

**DOI:** 10.1007/s00795-025-00425-3

**Published:** 2025-03-10

**Authors:** Takuma Miura, Arisa Watanabe, Mutsumi Miyake, Sayaka Suga, Makoto Miyoshi, Kumiko Miyashita, Shohei Komatsu, Noriyuki Nishimura, Kazuya Shimizu, Yuichi Hori

**Affiliations:** 1 Department of Biophysics, Kobe University Graduate School of Health Sciences, 7-10-2 Tomogaoka, Suma-Ku, Kobe, 654-0142 Japan; 2 Department of Nursing, Kobe University Graduate School of Health Sciences, Kobe, Japan; 3 Department of Surgery, Division of Hepato-Biliary-Pancreatic Surgery, Kobe University Graduate School of Medicine, Kobe, Japan; 4 Department of Internal Medicine, National Hospital Organization Kobe Medical Center, Kobe, Japan

**Keywords:** Pancreatic cancer, Patient-derived xenograft (PDX), Orthotopic transplantation, Metastasis, Trousseau’s syndrome

## Abstract

Pancreatic cancer is a malignant tumor that metastasizes to distant organs, such as the liver and lungs from an early stage. Few animal models can reproduce early metastasis. In addition, no model has been reported that reproduces cancer-related hypercoagulability, which is characteristic of pancreatic ductal adenocarcinoma and other adenocarcinomas. We hypothesized that the reason why the commonly used orthotopic cell xenograft model cannot reproduce the disease is inadequate construction of the cancer microenvironment. We developed an orthotopic tissue fragment xenograft model in which tumor tissue was transplanted into the pancreas of mice while preserving the microenvironment. Briefly, we injected single cancer cells subcutaneously to form a tumor, which was then cut with a scalpel into tumor fragments. A fragment was then sutured and fixed to the surface of the pancreatic tail. In this study, we evaluated the superiority of this model over a conventional orthotopic cell xenograft model. As a result, the novel orthotopic tissue xenograft model reproduced early distant metastasis to the liver and lung, nerve invasion, and cancer-related hypercoagulability of human pancreatic cancer, and showed greater similarity to clinical cases than the control orthotopic cell xenograft model.

**Supplementary Information:**

The online version contains supplementary material available at 10.1007/s00795-025-00425-3.

## Introduction

Pancreatic ductal adenocarcinoma (PDAC) is one of the most lethal malignancies, and despite advances in multidisciplinary treatment, the 5-year survival rate remains approximtely 10% [1]. Distant metastasis of PDAC is associated with a poor prognosis. Although ectopic (subcutaneous) patient-derived xenograft (PDX) and orthotopic (intrapancreatic) patient-derived xemograft (PDOX) models using pancreatic cancer cells have been used to elucidate the mechanism of distant metastasis, these models cannot efficiently reproduce distant metastasis. The main reason for this may be the difficulty of forming a cancer microenvironment during the observation period. In addition to cancer cells, tumor tissues contain vascular endothelial cells, immune cells, and fibroblasts, and the microenvironment is constructed by these cells. The microenvironment functions as a niche to control the maintenance, proliferation, invasion, and metastasis of cancer cells [2].

Genetically engineered mouse models expressing mutant KRAS and related genes specifically in the pancreas have been used as a model of pancreatic cancer, but this is not necessarily an ideal model in terms of microenvironment construction or distant metastasis [3]. In contrast, PDX models, such as ectopic subcutaneous xenografts, and PDOX models such as orthotopic xenografts, are also thought to mimic the pathophysiology and molecular properties of pancreatic cancer [4, 5]. Furthermore, the PDOX model recapitulates the tumor microenvironment more closely than the ectopic subcutaneous xenograft model and mimics liver metastasis [5]. Our laboratory has previously established cancer cells from patients with pancreatic cancer and generated ectopic subcutaneous xenograft (PDX) and orthotopic pancreatic xenograft (PDOX) models. However, they did not show distant metastases (e.g., liver and lung metastases), which are characteristic of pancreatic cancer [6, 7].

Recently, cancer-related hypercoagulopathy (so-called Trousseau’s syndrome) has become a major problem in patients with cancer [8, 9]. In particular, adenocarcinomas including pancreatic and lung cancers have been reported to be associated with a high risk of cancer-related hypercoagulopathy [9, 10]. However, none of the current pancreatic cancer models are appropriate for developing treatments for cancer-related thrombosis.

In this study, we established a novel orthotopic tissue xenograft model that maintains the microenvironment of cancer cells (patient-derived orthotopic tissue xenograft [PDOTX]) and compared it with a conventional orthotopic cell xenograft model (patient-derived orthotopic cell xenograft [PDOCX]) in terms of metastatic potential, nerve invasion, and coagulation capacity.

## Materials and methods

### Cells

Human pancreatic cancer cell lines (KMC34-GFP, KMC26-GFP) KMC34,26 cells were established from human pancreatic cancer patients in our laboratory [6, 7], and the green fluorescent protein (GFP) gene was introduced by retrovirus [7, 11]. KMC cells were cultured in serum-free Stem medium (DSRK100; DS Pharma Biomedical) containing 0.1 µM 2-mercaptoethanol, 50 U/mL penicillin-50 µg/mL streptomycin.

### In vivo transplantation experiments

**Fig. 1:**
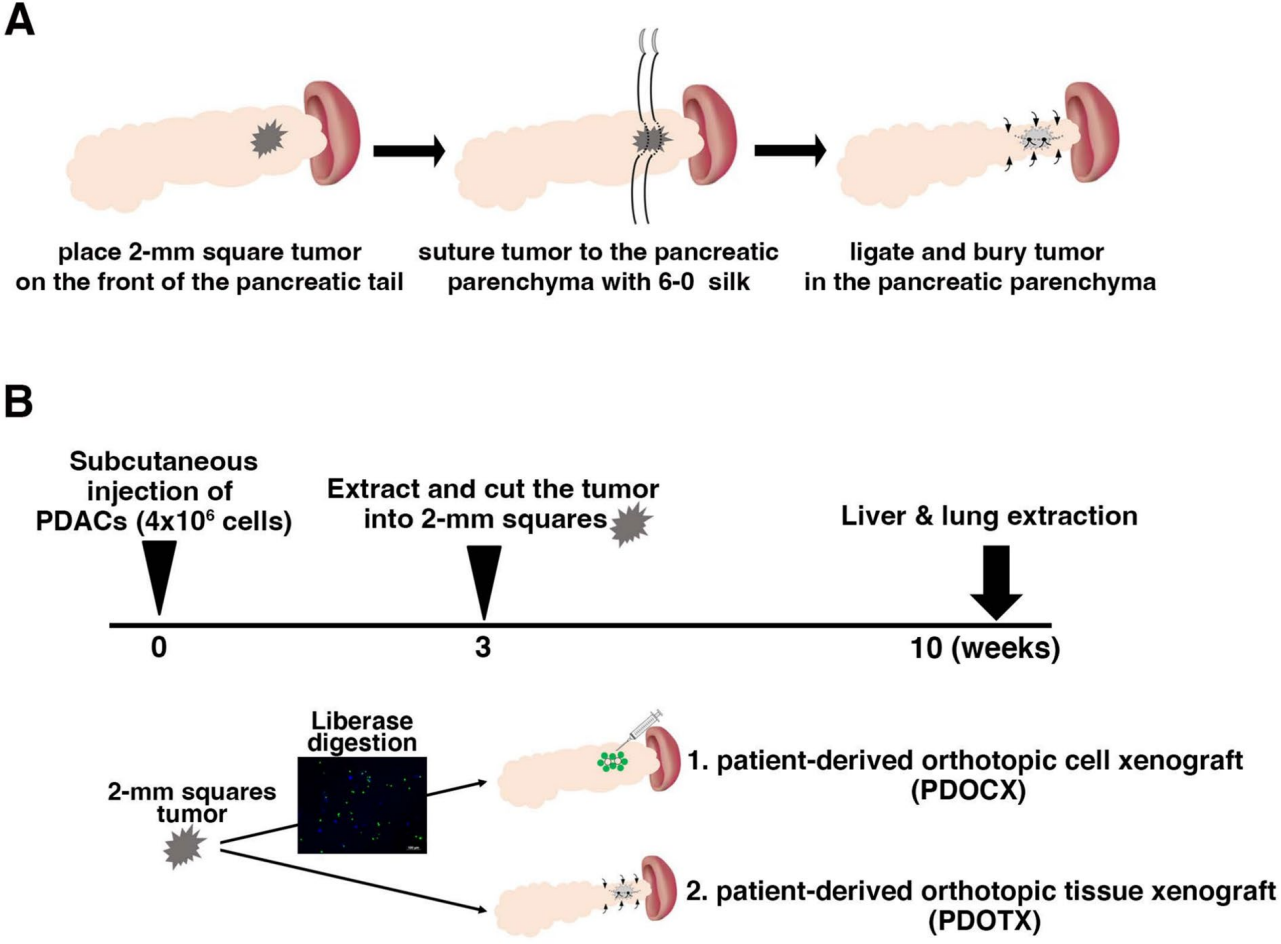
In vivo human pancreatic cancer cell transplantation experiments. **A** The procedure of establishing the patient-derived orthotopic cancer tissue xenograft model (PDOTX) using 8mm^3^ human pancreatic cancer cell (GFP-KMC34 or GFP-KMC26)-derived tumor. See also Materials and Methods, and Supplementary Video 1. **B** A schematic illustration of in vivo transplantation. First, we injected 4 × 10^6^ GFP-KMC34 or GFP-KMC26 cells subcutaneously into immunocompromised mice to form subcutaneous tumors. Three weeks later, we extracted and cut the tumor into 2-mm sequres (8 mm^3^). In the case of the conventional patient-derived orthotopic cancer cell model (PDOCX), 8 mm^3^ grafts were digested with Liberase Blenzyme 3 to single cells and whole cells were injected into the pancreatic tail of nude mice. On the other hand, the PDOTX model was established by suturing 8mm^3^ grafts to the pancreatic tail of nude mice, as shown in Fig. 1A. Seven weeks later, the grafts were extracted and analyzed

(1) KMC-GFP cells (4 × 10⁶) were mixed with Matrigel and transplanted subcutaneously into nude mice. After 3–4 weeks, the tumors were removed and cut into 2-mm cubes (8 mm^3^) to create grafts. In the PDOTX model, the graft (8 mm^3^) was sutured to the tail of the pancreas in nude mice (*n* = 5). The details are presented (Fig. 1A and Supplementary Video 1). The mouse was placed in the right lateral position under isoflurane inhalation anesthesia, and a transverse incision was made on the left chest. (2) A retroperitoneal incision was made parallel to the incision to avoid damaging the intercostal artery and vein. (3) A cotton swab was used to remove the spleen from the abdominal cavity, and detach the splenic and pancreatic ligaments with a cotton swab to free the spleen and tail of the pancreas. (4) The tumor fragment was placed in front of the pancreatic tail and fixed to the pancreas with a 6–0 suture to prevent the tumor fragment from deviating from the pancreas. (5) The tumor was covered with sutures to prevent it from being exposed to the pancreas and cancer cells from spreading into the abdominal cavity. (6) The tumor and tail of the pancreas were returned to the abdominal cavity, and the peritoneum with a 3–0 nylon thread. The skin was then closed using a stapler. Figure 1B shows a schematic illustration of in vivo transplantation. As for the PDOTX model, the tumor fragment was transplanted as described above and, the liver and lungs were removed and analyzed two months after transplantation. As a control (PCOCX) model, 8mm^3^ grafts were digested into single cells in phosphate-buffered saline (PBS) containing 0.04 units of Liberase Blenzyme 3 (Roche Diagnostic, Basel, Switzerland), suspended in 200 μl Gibco Dulbecco's Modified Eagle Medium (DMEM), and transplanted into the tail of the pancreas of nude mice (*n* = 5) using a 27G needle (PDOCX). The number of GFP-positive (KMC-GFP) and GFP-negative cell in 8 mm^3^ grafts was 1.4 × 10^5^ and 1.2 × 10^5^, respectively. At 2 months after transplantation, the liver and lungs were removed and analyzed as well. No mice died during the observation period.

### Immunohistochemical staining

After perfusion and deblooding in PBS, the liver and lungs were removed, fixed in 4% paraformaldehyde overnight, replaced with sucrose, and embedded in Optimal Cutting Temperature (Sakura Finetek) to prepare frozen sections. Frozen sections were air-dried and blocked with Blocking One Histo (Nacalai Tesque) for 10 min. The anti-GFP antibody (Abcam) was diluted 1:500 in 10% FCS in Tris-buffered saline (pH 7.6). containing 0.1% TritonX-100 (10% FCS/TBST) at a dilution ratio of 1:500, and incubated overnight at 4 °C. The secondary antibody was anti-chicken Alexa Fluor 488 (Invitrogen) diluted 1:200 in 10% FCS/TBST and incubated for 60 min at room temperature, followed by DAPI for 10 min at room temperature. In pancreatic primary tumors, anti-GFP antibodies (Abcam) and anti-peripherin antibodies (Invitrogen) were diluted 1:500 and 1:1000, respectively, as described above, and incubated overnight at 4 °C. DAPI was reacted with anti-chicken Alexa Fluor 488 (Invitrogen) at 1:200 and anti-rabbit Alexa Fluor 555 (Invitrogen), as a secondary antibody, at a dilution of 1:200 for 60 min at room temperature at the same dilution, as described above. The same procedure was performed as previously described. Images were obtained using a fluorescence microscope (ZEISS Axio Vert. A1). The following antibodies were used to evaluate metastasis-related gene products. Anti-HIF1α antibodies (Abcam), anti-Snail antibodies (Abcam), and anti-FOXM1 antibodies (Merck) were diluted 1:500, 1:200, 1:100, respectively.

### Sirius red staining

Tissue samples were fixed in formalin, embedded in paraffin, and sectioned. Sirius Red staining was performed according to the manufacturer's instructions (Picro-Sirius Red Stain Kit [ScyTek Laboratories]).

### Enzyme-linked immunosorbent assay (ELISA)

Plasma D-dimer and thrombin antithrombin III complex (TAT) concentrations were measured in the untreated, PDOTX, and PDOCX groups by the D-dimer ELISA (MyBioSource) and TAT ELISA (ASSAYPRO) kits, respectively.

### Statistical analysis

Statistical significance was determined using a two-tailed unpaired Student’s *t* test or *χ*^2^ test. (All parameters are expressed as mean ± standard error of the mean (SEM), and *P* values of < 0.05 were considered to induce statistical significance.

## Results

### Pancreatic tumor volume

**Fig. 2:**
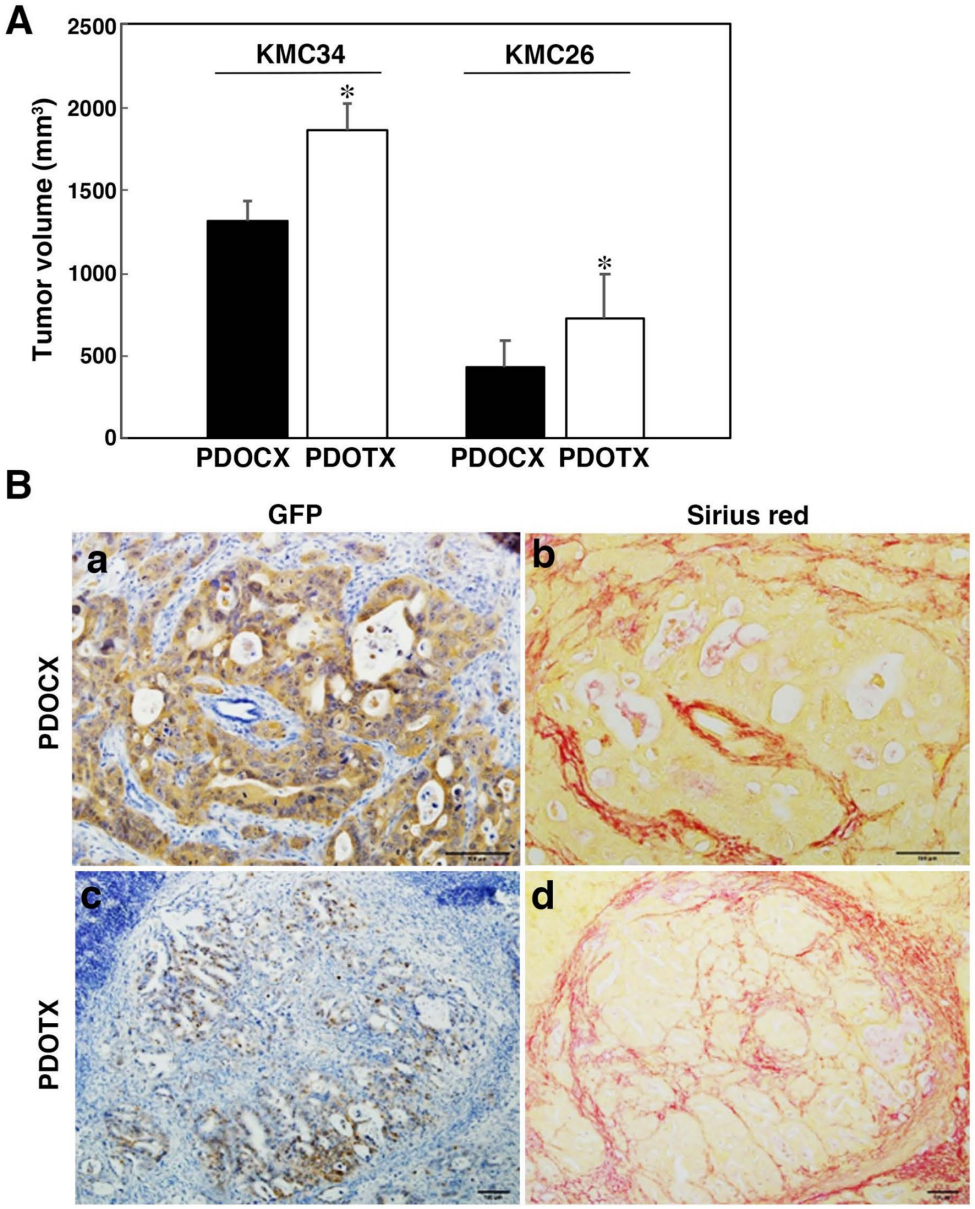
Comparison of PDOCX and PDOTX in tumor volume and stromal formation. **A** Tumor volume was significantly increased in the PDOTX model using KMC34 and KMC26 cells relative to the PDOCX model. **B** The histology of KMC-derived tumors (GFP staining) and stromal formation (Sirius red staining) in the PDOCX (**a**, **b**) and PDOTX (**c**, **d**) models, respectively. There was no clear difference in the ratio of stroma formation between the two groups. (*n* = 5; mean ± SEM; *, *P* < 0.05)

The size of tumors formed by tissue transplantation (PDOTX) and cell transplantation (PDOCX) was measured for KMC34 and KMC26, respectively, and it was confirmed that the tumor volume of the PDOTX group was significantly larger than that of the PDOCX group in both strains (Fig. 2A). However, there was no clear difference in the ratio of stroma formation between the two groups (Fig. 2B).

### Liver and lung metastasis

**Fig. 3:**
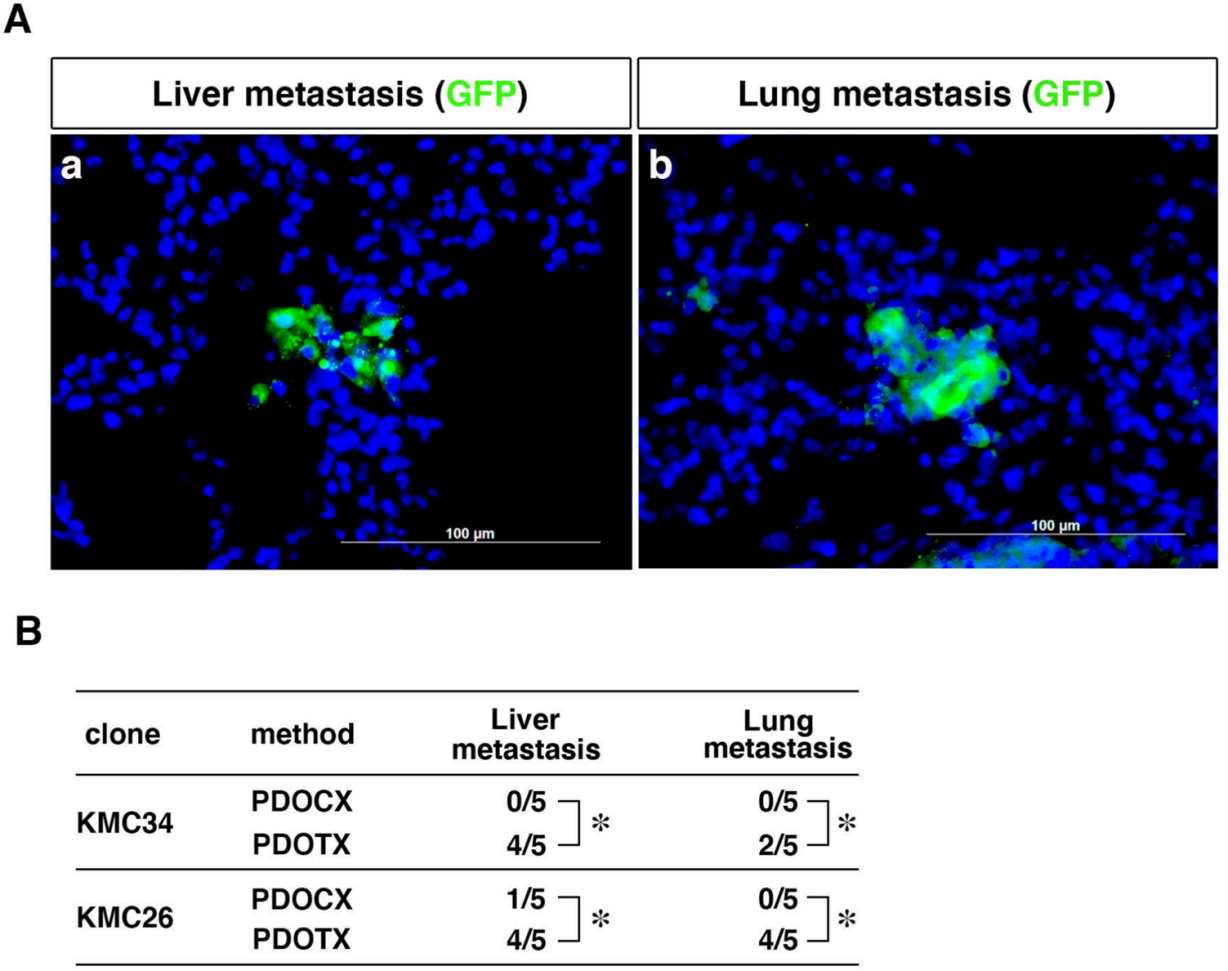
PDOTX model was superior to the conventional PDOCX model in promoting distant metastasis that was characteristic of human pancreatic cancer. **A** Immunofluorescence staining of (**a**) liver and (**b**) lung specimens using anti-chick GFP antibodies. Scale bar = 100 µm. **B** Summary of the frequency of distant metastasis to the liver and lungs. Distant metastasis to the liver and lungs was significantly enhanced in both KMC34 cells and KMC26 cells in the PDOTX model. (*n* = 5; *, *P* < 0.05 relative to the PDOCX model)

**Fig. 4:**
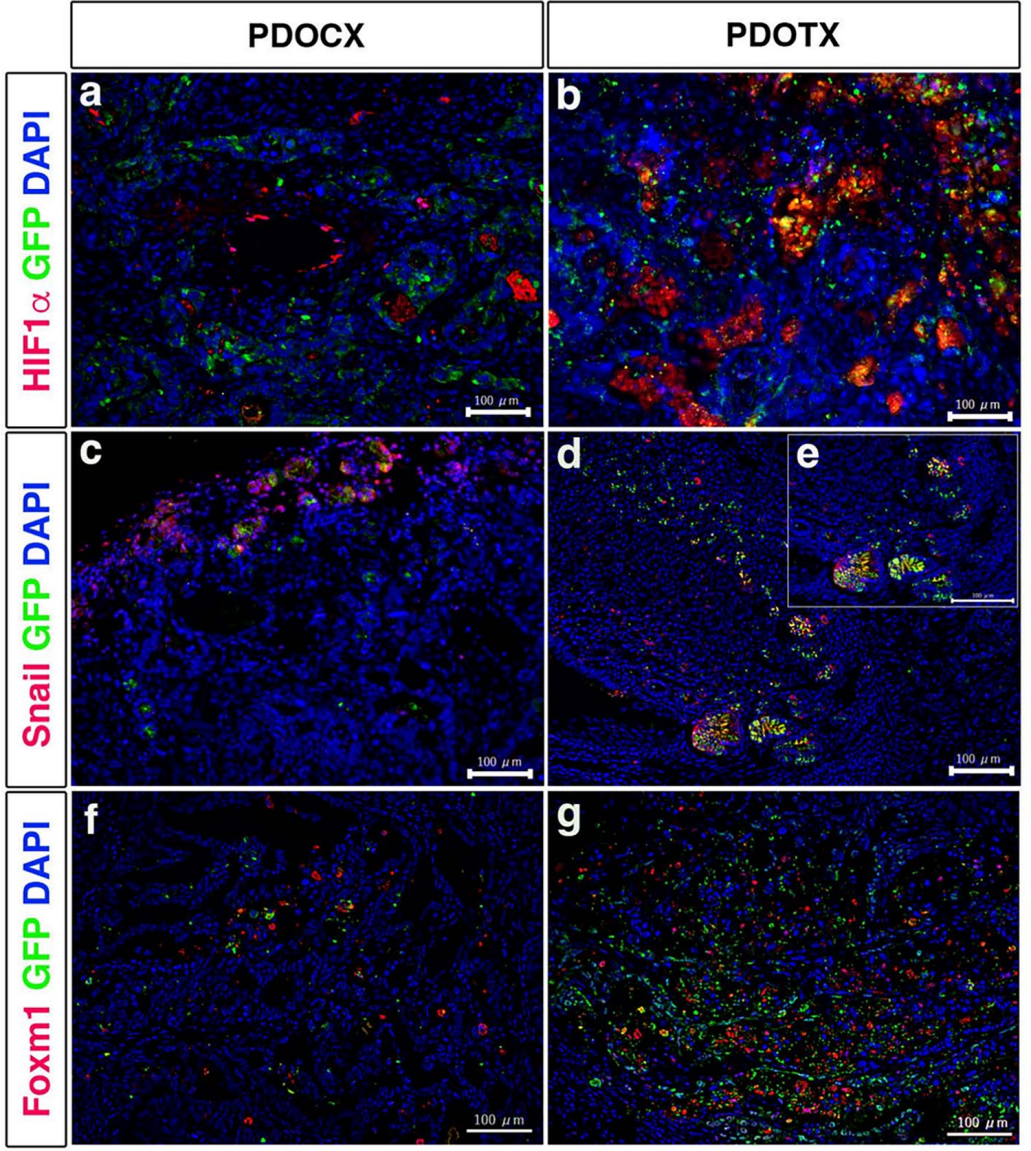
The expression of metastasis-related gene products in pancreatic tumors in PDOTX model was enhanced compared to those in the conventional PDOCX model. Immunofluorescence staining of **a** HIF-1α, **c** Snail, **f** FOXM1 in pancreatic tumors formed in PDOCX model. On the other hand, **b** HIF-1α, **d**, **e**: insel in **d** Snail, **g** FOXM1 in pancreatic tumors formed in the PDOTX model. Scale bar = 100 µm

With regard to metastasis to distant organs, in the model using KMC34 and KMC26 cells, no macroscopically apparent metastasis to the liver or lungs was observed in either the PDOCX or PDOTX groups. We have already used a similar method to show liver and lung metastases in the immunostaining of Figs. 2 and 4 in Kimoto et al.'s paper (Reference 18), together with GFP immunofluorescent staining. Histological GFP staining using KMC34 cells showed that 4 of the 5 mice in the PDOTX group had liver metastasis, and 2 of the 5 mice in the PDOTX group had lung metastasis. In contrast, no liver or lung metastases were observed in any of the 5 mice in the PDOCX group. Histological GFP staining using KMC26 cells showed that 4 of 5 mice in the PDOTX group had liver metastasis, and 4 of 5 mice in the PDOTX group had lung metastasis. In contrast, in the PDOCX group, liver metastasis was observed in 1 of the 5 mice, but no lung metastasis was observed (Fig. 3A, B). To evaluate metastasis-related gene products, we stained with HIF1α, Snail and FOXM1 antibodies. HIF-1α -Snail signaling pathway and FOXM1 are reported to induce epithelial-mesenchymal transition (EMT), which is involved in the metastasis and infiltration of pancreatic cancer. As a results, we found that the expression of metastasis-related gene products was enhanced in pancreatic tumors formed in the PDOTX group (Fig. 4b, d, e: insel in d), compared to pancreatic tumors formed in PDOCX group (Fig. 4a, c, f), suggesting that these results indicate that the PDOTX model may promote distant metastasis via EMT.

### Nerve invasion and lymph node metastasis

**Fig. 5:**
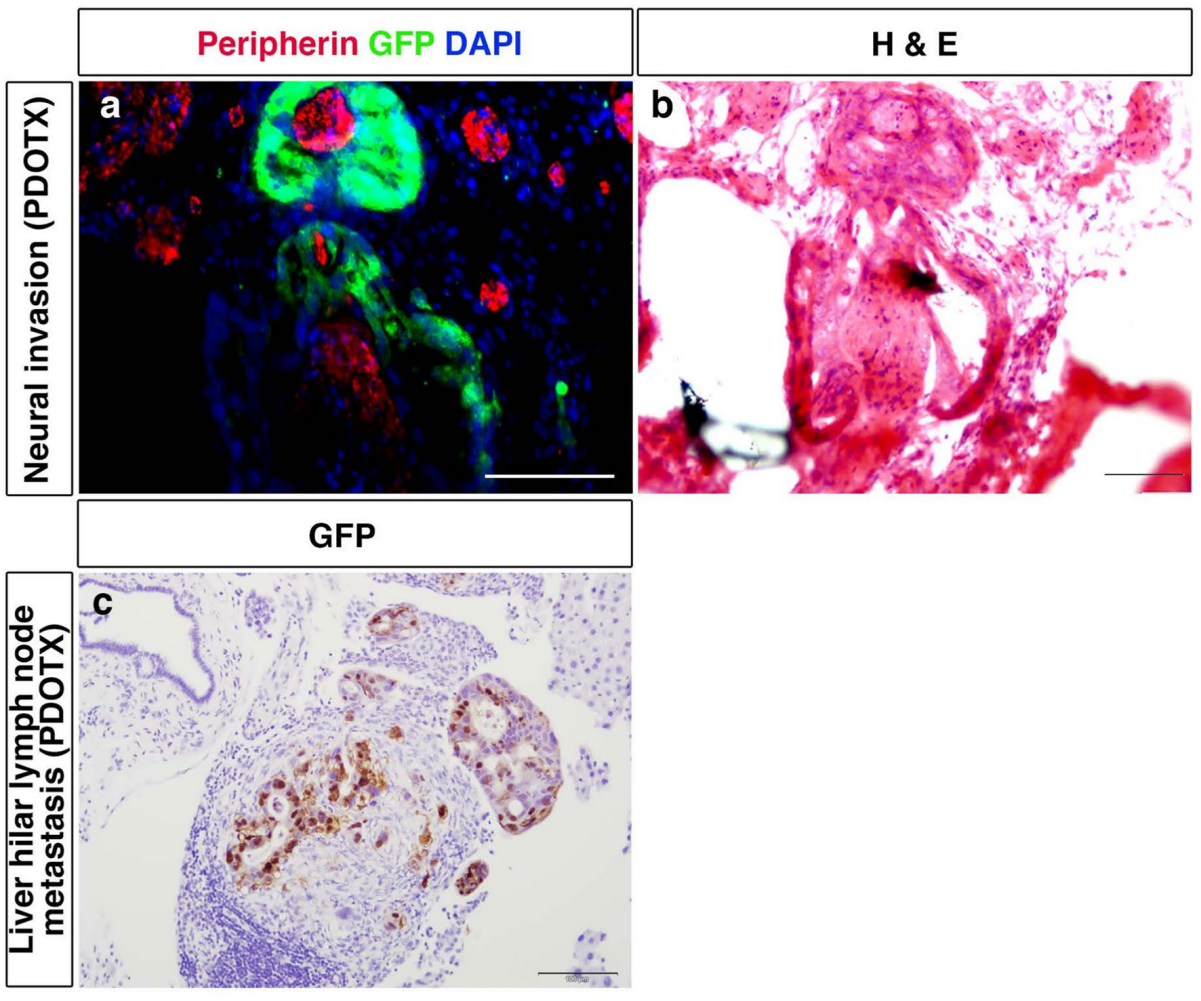
The PDOTX model but not PDOCX model, recapitulated the perineural invasion and lymph node metastasis that are characteristic of human pancreatic cancer. **a** Fluorescence immunostaining using an anti-Peripherin antibody to label the peripheral nerve showed peri-neural invasion by GFP-positive human pancreatic cancer cells. **b** H and E staining of the same area. **c** Liver hilar lymph node metastasis identified by GFP-staining

We examined nerve invasion, a characteristic of patients with pancreatic cancer When stained with peripherin, as a marker of peripheral nerves and GFP, we observed perineural invasion in the PDOTX group but not in the PDOCX group (Fig. 5a, b). Furthermore, gross lymph node metastasis in the hilar region was observed in the PDOTX group (Fig. 5c).

### Examination of blood hypercoagulability

**Fig. 6:**
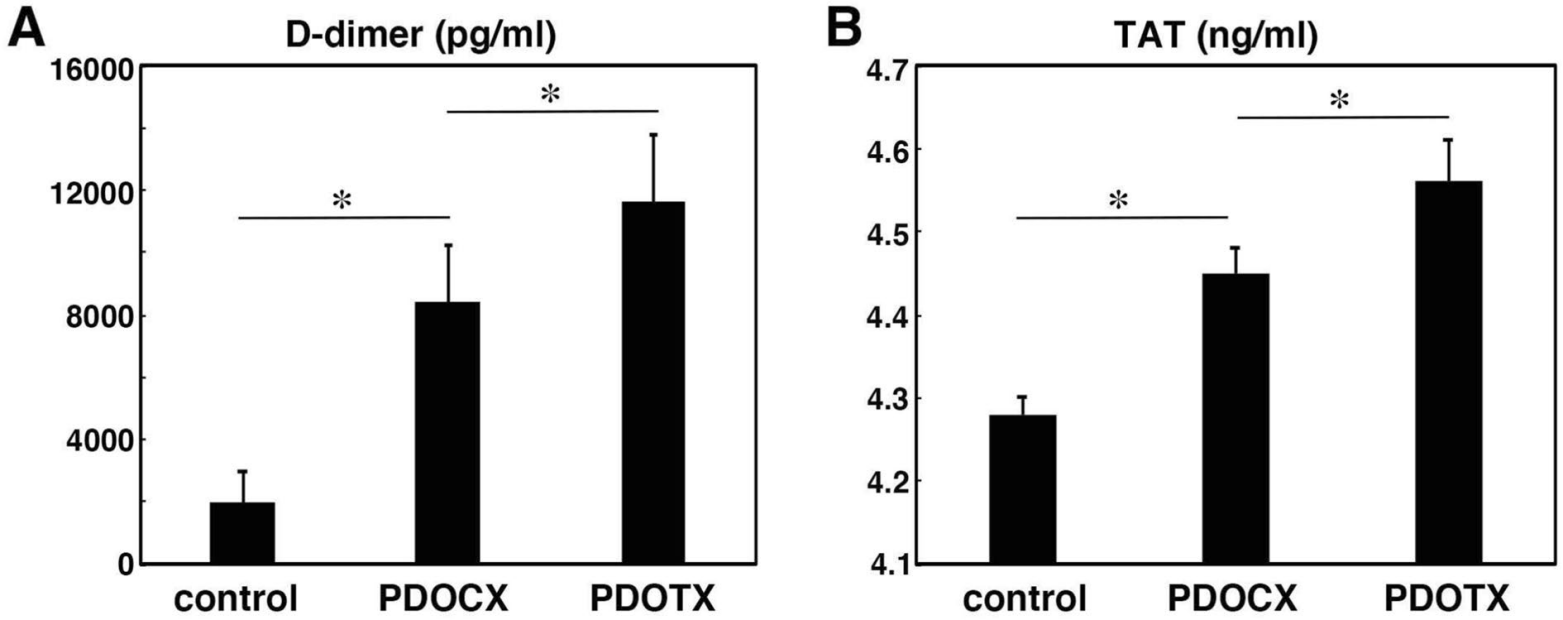
PDCTX model was superior to the conventional PDOCX model in promoting the cancer-related hypercoagulability that was characteristic of human pancreatic cancer. **a** Plasma D-dimer and **b** Plasma thrombin-antithrombin III complex (TAT) were significantly higher in the PDOTX model in comparison to control and the PDOCX model (*n* = 5; mean ± SEM; *, *P* < 0.05)

In malignant tumors, especially adenocarcinoma, Trousseau's sign, or hypercoagulability, is also related to the prognosis. Therefore, we measured plasma D-dimer and TAT levels using the ELISA method as indicators of coagulability and found that the PDOTX group had higher plasma D-dimer and TAT levels than the PDOCX group and the untreated control group, indicating that the PDOTX group had higher hypercoagulability (Fig. 6).

## Discussion

To date, two xenograft experimental models of human pancreatic cancer have been used: the patient-derived xenograft (PDX) model, represented by subcutaneous transplantation, and the patient-derived orthotopic xenograft (PDOX) model, represented by pancreatic transplantation. In the PDX model (mainly subcutaneous transplantation in mice), the transplant site does not coincide with the original site of tumor development, which is useful for determining the antitumor effect of the drug: however, the neural invasion and stromal formation characteristics of pancreatic cancer are not observed [12]. On the other hand, the PDOX model has the advantage that the microenvironment of pancreatic cancer is aligned, and metastasis in the PDOX model is enhanced in comparison to the PDX model. Furthermore, it has been reported that the sensitivity to gemcitabine was altered when the PDX model was converted to PDOX [5]. That is, the analysis of the gene expression, including the genes involved in the uptake and activation of gemcitabine, showed clustering in PDOX that was not observed in the primary tumor or PDX [13]. On the other hand, Go et al. prepared 2-mm square tumor fragments by orthotopic pancreatic transplantation, as in the present study, and examined the invasion of cancer into the pancreatic parenchyma, distant metastasis, and muscle wasting in a control PDX model in which the tumor was transplanted into the flank of a mouse. They reported that the orthotopically transplanted tumor fragment model induced muscle wasting in direct proportion to tumor size, consistent with cancer cachexia syndrome [14]. Although the detailed differences between our transplantation method and theirs are not clear, the most different result is that in the model reported by Go et al., all 13 of the mice showed distant metastasis to the lungs, whereas liver metastasis was only observed in 1 mouse [14]. In our model, liver metastasis was observed in 8 of 10 cases, in 2 clones (KMC34 and KMC26), while lung metastasis was observed in 6 of 10 cases. In clinical cases of pancreatic cancer, the liver is the most common site of metastasis, followed by peritoneum and the lung [15]; therefore, this model is considered closer to clinical cases. GFP staining demonstrated the presence of liver and lung metastases in the PDOTX model, whereas they were hardly observed in the PDOCX model. Recently, we reported that exosomes derived from human pancreatic cancer cells promote initial metastasis to the liver and lungs [16, 17], and it is possible that exosomes derived from pancreatic cancer cells are also involved in the superiority of the PDOTX model. Although there are many unknowns regarding the early stages of distant metastasis in clinical cases and in vivo models, because the tumor microenvironment is maintained in PDOTX, it is thought that the tumor cells of the transplanted tumor fragments infiltrated the surrounding area, causing distant metastasis, neural invasion, and lymph nodes metastasis in the hepatic hilum via the portal vein and lymph vessels. There was no significant difference in stroma formation between the PDOTX and PDOCX groups, but the reason for this is currently unknown.

From the viewpoint of cardiovascular oncology, Trousseau’s syndrome or cancer-associated hypercoagulability, which has increasingly become a concern among cancer patients in recent years, serves as a typical example of a tumor-associated syndrome that poses a significant risk to patients with adenocarcinomas of the pancreas, stomach, and lung [9, 10]. There is a report of a patient who had multiple cerebral infarctions and was subsequently diagnosed with Trousseau’s syndrome associated with lung cancer after a systemic examination, in which FDP, D-dimer, and TAT levels were found to be elevated on admission, and the patient was diagnosed with Trousseau’s syndrome due to abnormal coagulation associated with lung cancer [18, 19]. In the PDOCX model, both plasma D-dimer and TAT levels were elevated in comparison to those in the normal control group, demonstrating that this model reproduced the hypercoagulable state. Furthermore, in the PDOTX model, plasma D-dimer and TAT levels were even higher than in the PDOCX model, indicating that this model is a better treatment model for cancer-associated hypercoagulability. In the PDOCX and PDOTX models, the elevated plasma D-dimer and thrombin-antithrombin III complex (TAT) levels likely reflect the presence of systemic coagulability that is characteristic of cancer, including pancreatic cancer. In particular, the hypercoagulable state in these models could be due to several factors:

Tumor-derived pro-coagulant factors: Pancreatic cancer cells, including those used in our models (KMC34 and KMC26), may secrete pro-coagulant molecules, such as tissue factor (TF), that trigger the coagulation cascade. This is consistent with findings from other cancer models, where tumor cells promote the activation of coagulation via TF and other factors, leading to thrombin generation and fibrin formation.

Inflammation and immune response: The tumor microenvironment in both PDOCX and PDOTX models likely involves a significant inflammatory response, which can also contribute to hypercoagulability. Tumor-associated inflammation promotes the release of not only cytokines and inflammatory mediators, but also secreted exosomes which may indirectly enhance coagulation pathways. Additionally, interactions between tumor cells and immune cells may activate platelets and other clotting factors, further exacerbating the hypercoagulable state.

Direct oral anticoagulants (DOACs), a new class of oral anticoagulants for deep vein thrombosis, have also been approved for the treatment of Trousseau syndrome [18, 20]. DOACs are attracting attention because they are as effective as the conventionally used warfarin and heparin and carry a lower risk of bleeding: however, the evidence on the efficacy of DOACs in Trousseau syndrome is still insufficient in pancreatic cancer [21, 22]. We believe that the model we developed may be useful for future studies to investigate optimal drugs for Trousseau's syndrome. Although we developed a model that reproduces distant metastasis, nerve invasion, and hypercoagulability, there is room for further investigation of these mechanisms. Although our novel PDOTX model demonstrates a more accurate reproduction of the metastatic potential, nerve invasion, and hypercoagulability seen in clinical cases of pancreatic cancer, several limitations should be considered.

1. Immunodeficient Mouse Model: The PDOTX model was conducted in nude mice, which are immunodeficient. This limits our ability to evaluate how the immune system interacts with the tumor microenvironment and contributes to metastasis or hypercoagulability. Future studies using immunocompetent models or humanized mice would be beneficial to better understand the role of immune cells in the progression of PDAC. We will extend our investigations to immunocompetent mouse models to evaluate immune-tumor interactions.

2. Longitudinal Validation: Our study provides valuable initial evidence for the relevance of the PDOTX model, but further longitudinal studies examining long-term tumor progression, metastasis, and therapeutic responses would strengthen the model's clinical relevance. Additionally, studies to assess the model's response to treatment with different types of chemotherapy, targeted therapies, or anticoagulants would be crucial for assessing its potential in drug testing. We will explore the therapeutic potential of the PDOTX model by evaluating its response to various cancer treatments.

We hope that the discussion of these limitations and plans for further research will clarify the need for additional studies to confirm and extend our findings.

## Conclusion

We established the PDOTX model, a novel orthotopic tissue xenograft model that more accurately recapitulates the metastatic potential, nerve invasion, and hypercoagulability seen in clinical cases of pancreatic cancer compared to the conventional orthotopic cell xenograft model (PDOCX).

## Supplementary Information

Below is the link to the electronic supplementary material.

Supplementary file1 (MPG 95174 KB)

## Data Availability

The datasets generated and analyzed during the current study are available from the corresponding author on reasonable request.
